# Distinct Effects of Rotenone, 1-methyl-4-phenylpyridinium and 6-hydroxydopamine on Cellular Bioenergetics and Cell Death

**DOI:** 10.1371/journal.pone.0044610

**Published:** 2012-09-06

**Authors:** Samantha Giordano, Jisun Lee, Victor M. Darley-Usmar, Jianhua Zhang

**Affiliations:** 1 Department of Pathology, Center for Free Radical Biology, University of Alabama at Birmingham, Birmingham, Alabama, United States of America; 2 Department of Veterans Affairs, Birmingham VA Medical Center, Birmingham, Alabama, United States of America; Emory University, United States of America

## Abstract

Parkinson’s disease is characterized by dopaminergic neurodegeneration and is associated with mitochondrial dysfunction. The bioenergetic susceptibility of dopaminergic neurons to toxins which induce Parkinson’s like syndromes in animal models is then of particular interest. For example, rotenone, 1-methyl-4-phenyl-1,2,3,6-tetrahydropyridine (MPTP) and its active metabolite 1-methyl-4-phenylpyridinium (MPP^+^), and 6-hydroxydopamine (6-OHDA), have been shown to induce dopaminergic cell death *in vivo* and *in vitro*. Exposure of animals to these compounds induce a range of responses characteristics of Parkinson’s disease, including dopaminergic cell death, and Reactive Oxygen Species (ROS) production. Here we test the hypothesis that cellular bioenergetic dysfunction caused by these compounds correlates with induction of cell death in differentiated dopaminergic neuroblastoma SH-SY5Y cells. At increasing doses, rotenone induced significant cell death accompanied with caspase 3 activation. At these concentrations, rotenone had an immediate inhibition of mitochondrial basal oxygen consumption rate (OCR) concomitant with a decrease of ATP-linked OCR and reserve capacity, as well as a stimulation of glycolysis. MPP^+^ exhibited a different behavior with less pronounced cell death at doses that nearly eliminated basal and ATP-linked OCR. Interestingly, MPP^+^, unlike rotenone, stimulated bioenergetic reserve capacity. The effects of 6-OHDA on bioenergetic function was markedly less than the effects of rotenone or MPP^+^ at cytotoxic doses, suggesting a mechanism largely independent of bioenergetic dysfunction. These studies suggest that these dopaminergic neurotoxins induce cell death through distinct mechanisms and differential effects on cellular bioenergetics.

## Introduction

Parkinson’s disease is the second most common neurodegenerative disease, affecting over 4 million people with pronounced degeneration of the dopaminergic neurons of the substantia nigra [1]. Although genetic factors contribute to the disease, over 90% of Parkinson’s disease cases do not have an identified genetic cause [1]. Mitochondrial dysfunction has been proposed to play a major role in Parkinson’s disease pathogenesis and can be induced by both exogenous and endogenous neurotoxins [2]. The mitochondrial enzyme which has been most frequently implicated in Parkinson’s disease is complex I [2]. Dysfunction of this complex has been shown in mitochondria isolated from postmortem brains, skeletal muscle and platelets of Parkinson’s disease patients [3]–[10]. Cybrid cell lines with mitochondria from Parkinson’s disease patients also exhibit decreased complex I activity [9], [11]–[14].

To investigate Parkinson’s disease pathogenesis and to test for potential therapeutics, chemicals that cause dopaminergic toxicity have been used in a variety of cell-based and animal models [15], [16]. The most frequently studied compounds are structurally diverse, and include rotenone, MPTP (1-methyl-4-phenyl-1,2,3,6-tetrahydropyridine) and its active metabolite MPP^+^ (1-methyl-4-phenylpyridinium) and 6-hydroxydopamine (6-OHDA) [15], [16]. All have been shown to inhibit mitochondrial complex I, either directly or indirectly, in assays involving isolated mitochondria [17]–[29]. Whether the extent of cell death induced by these toxins is directly related to their impact on mitochondrial function has not been assessed. The examination of bioenergetic function in intact cells is important because experiments with isolated mitochondria are typically constrained to a very narrow range of conditions including saturating concentrations of respiratory substrates or ADP, that do not occur in a cellular context. An understanding of the cellular effects of rotenone, MPP+ and 6-OHDA is also potentially important to better understand the gene-environment interactions in the context of Parkinson’s disease.

Rotenone has been used as an insecticide or fish poison for the past 50–150 years and has been studied as a potential cause of PD. Mixed reports have been published, and a conclusion regarding a causative role correlating with the dose and duration of rotenone exposure in PD is difficult to reach, due to insufficient longitudinal tracking, heterogeneity and combinatorial natural of environmental exposures [30]. Meta-analyses examining 19, 39 or 59 studies have concluded that pesticide exposures potentially increase PD risk on average ∼1.5–3 fold [31]–[33]. The recent and by far the most rigorous study by Tanner et al. reported that exposure to paraquat and rotenone by farming communities increased the incidence of PD with a odds ratio of ∼2–3 fold [34]. Rotenone is able to pass the blood brain barrier and plasma membrane, and radiolabeled [3H]dihydrorotenone binds to striatal sections from rodent brains with a Kd of ∼55 nM [35]. Although rotenone can freely diffuse into cells due to its hydrophobicity, in animal models, dopaminergic neurons appear to be particularly susceptible to rotenone-induced degeneration, [36]. Rats injected with 3 mg/kg of rotenone via subcutaneous osmotic minipump exhibit dopaminergic neurodegeneration in the nigrostriatal pathway and cytoplasmic α-synuclein aggregates in nigra neurons [37]. *In vitro*, 4 weeks of 5 nM rotenone exposure induces both soluble and insoluble α-synuclein accumulation, increased caspase activation and apoptosis [38]. In differentiated SH-SY5Y cells, 50 nM rotenone for 7 days induces Lewy neurite-like structures [39].

MPTP first associated with an increased incidence of Parkinsonism in an uncharacteristically young patient population [40], [41]. MPTP is converted into its active metabolite, MPP^+^, which is selectively taken up by dopaminergic cells via the dopamine transporters and induces dopaminergic cell death in mice, rats and primates [15], [16], [42]. Overexpression of the dopamine transporter into cells can change the susceptibility of cells to MPP^+^ toxicity. For example, the dopamine transporter has been expressed COS, HeLa and neuroblastoma SK-N-MC cells and this decreases the concentration of MPP^+^ needed to cause toxicity [43], [44]. The differentiated neuroblastoma cells we have used in this study are an established model to evaluate neurotoxicity, because these cells exhibit neurite extension, markedly decreased cell division and expression of neuronal markers [45], [73], [76], [77], [81], [82]. Retinoic acid was used as the differentiating agent because it results in transport characteristics for dopamine (Vmax of 21 pmol/mg protein, Km of 45 nM) which are similar to those reported for rat striatal synaptosomes (Vmax of 33 pmol/mg protein, Km of 29 nM) [45].

MPP^+^ is reported to inhibit rat or mouse mitochondrial pyruvate oxidation with Ki ranging from 60 to 400 µM, about 1000 fold higher than rotenone [19]. The weak inhibitory effect of MPP^+^ on complex I raised questions regarding its mechanisms of toxicity. For example, MPP^+^ at a concentration of 200 µM can induce partial and transient inhibition of complex III and IV activities in mitochondria from mouse brains [46]. In dopaminergic LUHMES cells, MPP^+^ depletes cellular ATP at the low concentration of 5 µM consistent with a bioenergetic mechanism distinct from the isolated mitochondria and which cannot be simply explained by increased transport into the cells [47]. Furthermore, in mesencephalic dopaminergic neurons, MPP^+^ inhibits mitochondrial trafficking at 2 µM which led us to the hypothesis that the interaction of MPP^+^ with cellular bioenergetic mechanisms may be distinct from those with isolated mitochondria {Kim-Han, 2011 5257/id}. Another mechanism that could contribute to MPP^+^ toxicity is oxidative stress. In support of this adding cellular or exogenous antioxidants has been shown to partially attenuate the detrimental effects of MPP^+^ {Przedborski, 1992 5272/id;Klivenyi, 1998 5271/id}.

The idea that both rotenone and MPP^+^ bind and inhibit complex I has been supported by the observation that exogenous expression of the yeast rotenone resistant complex I subunit NDI1 attenuated both rotenone and MPP^+^ toxicity *in vitro* and *in vivo* [29], [51]–[55]. Interestingly, NDI1 attenuated rotenone inhibition of cell growth, but did not attenuate cell growth in MPP^+^ treated cells unless under conditions of glucose deprivation, indicating different effects of the two compound [55]. These data suggest that the effects of MPP^+^ on cellular bioenergetics are more complex than simple inhibition of complex I. The obligatory role of complex I inhibition as a mechanism of rotenone toxicity was further challenged by a recent study showing that the absence of a complex I subunit Ndufs4, did not change the susceptibility to rotenone or MPP^+^-induced cytotoxicity, even though complex I activity is decreased [56]. Taken together these data highlight a persistent and interesting controversy in the literature regarding the impact on bioenergetics of rotenone and MPP^+^.

6-OHDA has been found in human brain [57] and human urine, with elevated levels in the urines of Parkinson’s disease patients who were treated with L-dopa [58]. Systemic injection to rodents caused depletion of norepinephrine in the heart {Porter, 1965 5267/id;Porter, 1963 5268/id}, while stereotaxic injection to the striatum induced degeneration of dopaminergic neuron processes in the striatum and dopaminergic neuron death in the substantia nigra [61]. 6-OHDA has been shown to reversibly inhibit both complexes I and IV activities in rat brain mitochondria with IC50 of ∼10 and 34 µM [26]–[28]; while it can also be a source of both hydrogen peroxide and superoxide radical through an auto-oxidation reaction [62], [63]. *In vitro* it has been shown that dopamine reacts with Fe(II) in the presence of hydrogen peroxide, to generate 6-OHDA [64]. 6-OHDA can then react with Fe(III) causing its release and subsequent cellular damage [65]. This is potentially important for PD pathogenesis, because the dopaminergic neurons of the substantia nigra in human brains have higher iron levels associated with the protein neuromelanin [66], [67]. Whether the effects of 6-OHDA on cell survival are related to damage to mitochondria in a cellular context is not known.

In summary, the effects of rotenone, MPP^+^ and 6-OHDA on activities of respiratory complexes have been investigated in isolated mitochondria with substrates and ADP in excess, but their effects on bioenergetic function and the relationship to toxicity in intact cells remains unknown. A novel approach to assessing cellular bioenergetics is to use extracellular flux analysis which uses real time measurement of oxygen consumption and pH in adherent cells [68]–[70]. This technology overcomes many of the drawbacks inherent in experiments with isolated mitochondria, because it allows the cell to provide physiologically relevant levels of respiratory substrates and ADP [68]–[70]. Because both rotenone and MPP^+^ are reversible inhibitors, accurately determining bioenergetic dysfunction from mitochondria isolated after exposure to these compounds is not feasible. The extracellular flux analysis also overcomes this problem. Using this approach we were able to relate the toxicity of the three toxins to their effects on cellular bioenergetics. At concentrations inducing a similar level of toxicity distinct effects on cellular bioenergetics were observed.

## Methods

### Cell Culture

Early passage P8-17 human neuroblastoma SH-SY5Ycells, grown in DMEM supplemented with 10% fetal bovine serum, 2 mM Glutamine, and penicillin/streptomycin were used. Differentiation was induced as described previously [70]. For bioenergetic measurements SH-SY5Y cells were grown in XF24 plates, differentiated with retinoic acid. These cells have extended axons, express the neuronal MAP2 marker, and have elongated mitochondria [70]. Both undifferentiated and differentiated (induced by various compounds) SH-SY5Y cells express dopamine transporters (DAT), and are susceptible to rotenone, MPP^+^ and 6-OHDA-induced cell death [45], [71]–[83]. We chose to use differentiated cells because they exhibit markedly decreased cell proliferation, many characteristics of neuronal cells and a dopamine transport system with similar characteristics to those synaptosomes [45].

### Measurement of Mitochondrial Function

To measure mitochondrial function in differentiated SH-SY5Y cells, the Seahorse Bioscience XF24 Extracellular Flux Analyzer (XF24) was used. The XF24 creates a transient, 7 µl chamber in specialized microplates that allows for the determination of oxygen concentration and pH in real time [69], [70]. Both the oxygen consumption rate (OCR) in pmol/min and the extracellular acidification rate (ECAR) in mpH/min, were normalized to total protein amount in individual wells determined by the DC protein assay (BioRad). The optimal seeding density of the cells needed to obtain a measurable O_2_ consumption rate and extracellular acidification rates (OCR and ECAR respectively) was established, and both ECAR and OCR show a linear response with cell number [70]. For subsequent experiments, a seeding density of 80,000 cells per well was selected to allow both potential increases and inhibition of OCR and ECAR to be assessed. Over the course of these experiments we found that the initial OCR prior to injection of compounds varied between preparations from 7–12 pmol/min/µg protein depending on small changes in cell culture conditions as we have reported previously for SH-SY5Y cells [70]. To allow comparison between multiple experiments data for OCR measurements are expressed as % of the stable rate prior to injection of neurotoxins. Mitochondrial function was assessed using the sequential injection of oligomycin, carbonyl cyanide 4-(trifluoromethoxy) phenylhydrazone (FCCP); and antimycin A concentrations to elicit maximal effects, which were optimized prior to assessment of bioenergetic function, and found to be 1 µM, 1 µM and 10 µM respectively. Rotenone was freshly prepared in DMSO and diluted into the medium, MPP^+^ was freshly prepared in the medium, and 6-OHDA was freshly prepared in 0.1% ascorbic acid. All experiments were performed with appropriate vehicle controls. The purity of MPP^+^ was assessed by mass spectrometry and a single peak detected at the anticipated molecular weight of 170.2 with no detectable impurities (result not shown). During these experiments either rotenone, MPP^+^, or 6-OHDA were injected after 4 baseline measurements at the beginning of the experiment, and OCR and ECAR were continuously measured for 2 hr, followed by a mitochondrial function assay described above and in detail in [69], [70].

### Cell Viability

Cell viability was measured first by Calcein AM assay and then confirmed using the trypan blue exclusion method. For the Calcein AM assay, 100,000 cells per well were plated and differentiated in 48-well plate. Calcein AM stock (1 mM) was prepared in 15 ml of Locke’s buffer. Media was removed from the cells and the cells were incubated for 30 min at 37°C with 150 µl Calcein AM in Locke’s Buffer. The cells were imaged on a Perkin Elmer Life Sciences Wallace 1420 multilabel plate reader. Green fluorescence was imaged with 488 nm excitation and 530 nm emission. For trypan blue exclusion assay, 150,000 cells were plated per well of a 12-well plate. Cells were treated with the neurotoxins for 24 hr and upon completion of exposure, the cells were trypsinized and mixed with trypan blue. Cells that excluded trypan blue were considered viable.

### Western Blot Analysis

300,000 cells were grown and differentiated in 6-well plates and treated for either 2 hr or 24 hr with different doses of rotenone, MPP^+^ and 6-OHDA. Protein extracts were separated by SDS-PAGE and probed with respective antibodies. Anti- caspase 3 antibody was from Cell Signaling (#9661), and actin antibody was from Sigma (#1978). Relative levels of protein were quantified using Image J software from the NIH (Bethesda, MA, USA).

### Statistical Analysis

Data are reported as mean ± SEM. Comparisons between two groups were performed with unpaired Student’s *t-*tests. One-way or Two-Way Analysis of Variants (ANOVA) was performed when multiple comparisons were made whenever appropriate. A *p* value of less than 0.05 was considered statistically significant.

## Results

### Effects of Rotenone, MPP^+^ and 6-OHDA on Caspase 3 Activation and Cell Death

**Figure 1:**
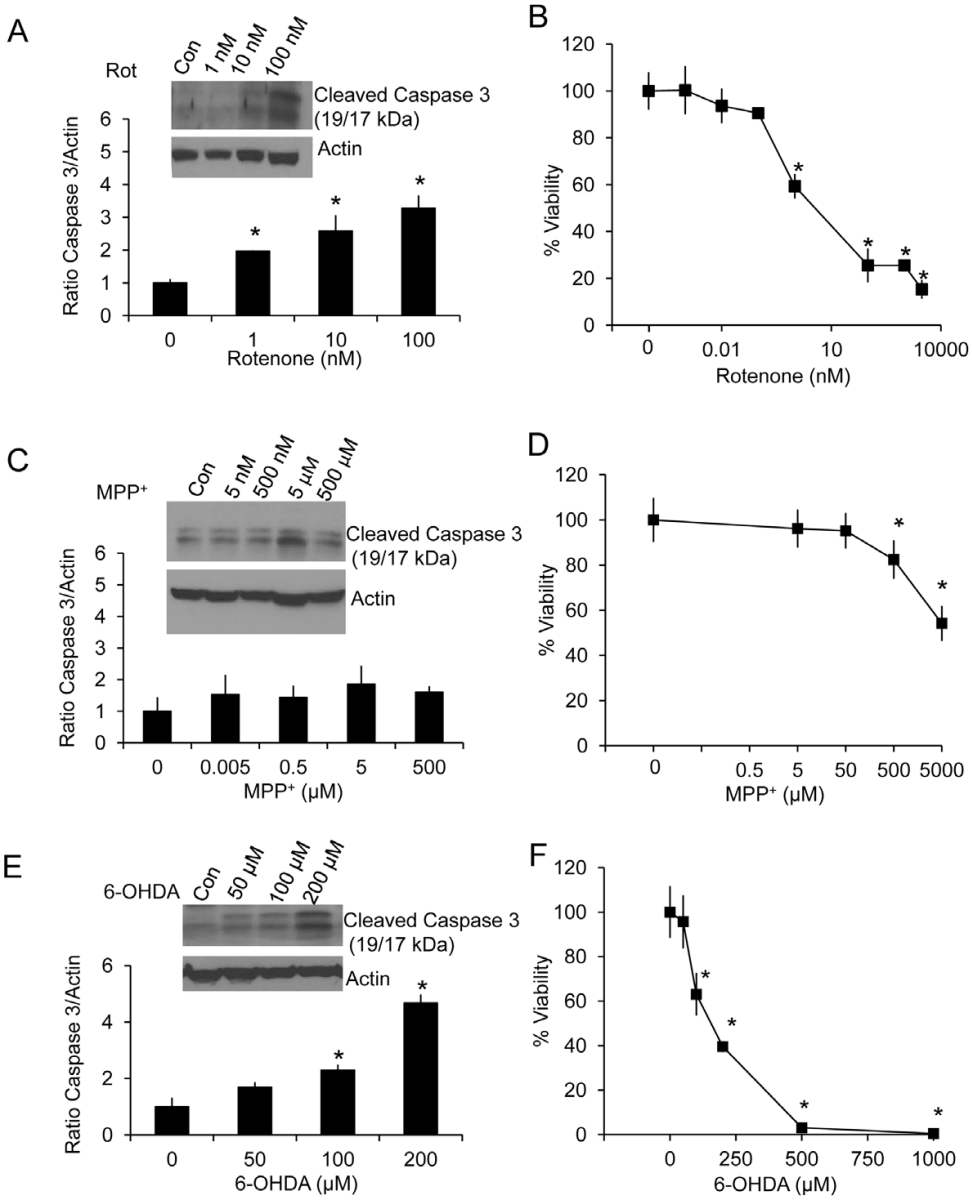
Caspase 3 activation and cell viability in response to rotenone, MPP^+^ and 6-OHDA. Whole cell lysates were collected after 24 hr exposure with increasing concentrations of rotenone, MPP^+^ and 6-OHDA. Western blot analysis for activated caspase 3 was performed using actin as a loading control, for increasing concentrations of rotenone (A), MPP^+^ (C), and 6-OHDA (E). Cell viability was assessed by trypan blue exclusion for rotenone (B), MPP^+^ (D), and 6-OHDA (F) after 24 hr exposure. Data are expressed as percent normalized to 0 µM treatment. Data  =  mean ± SEM, n = 3. *p<0.05, Student *t*-test compared to 0 µM treatment.

Differentiated human neuroblastoma SH-SY5Y cells were treated with increasing concentrations of rotenone, MPP^+^ and 6-OHDA. Western blot analyses of activated caspase 3 were performed at both 2 and 24 hr after the addition of the toxins. After 2 hr of various doses of the neurotoxins, no caspase activation was observed at any of the concentrations used (result not shown). After 24 hr of exposure to these toxins, the levels of activated caspase 3 increased progressively with increasing concentrations of rotenone. An increase of activated caspase 3 in response to MPP^+^ was not evident at any concentration tested. Increases of activated caspase 3 in response to 6-OHDA occurred at 100–200 µM concentrations (Figure 1A,C,E).

We determined cell viability in response to these neurotoxins by the trypan blue exclusion method. After 2 hr of exposure, no cell death was observed at any of the concentrations used (result not shown). At 24 hr rotenone was found to be the most potent inducer of cell death with 50% cell death induced at approximately 5 nM, MPP^+^ induced 50% cell death at ∼ 5 mM, and 6-OHDA induced 50% cell death at ∼ 100 µM (Figure 1B,D,F).

### Effects of Rotenone, MPP^+^ and 6-OHDA on Cellular Bioenergetics

To investigate the early cellular bioenergetic responses to rotenone, MPP^+^ and 6-OHDA, we performed studies using the XF24 analyzer. Initially a stable baseline for OCR was established for 32 min, at which point the compounds were injected directly onto the cells in the XF24 analyzer chamber, and the changes in OCR were monitored for a further 2 hr.

**Figure 2:**
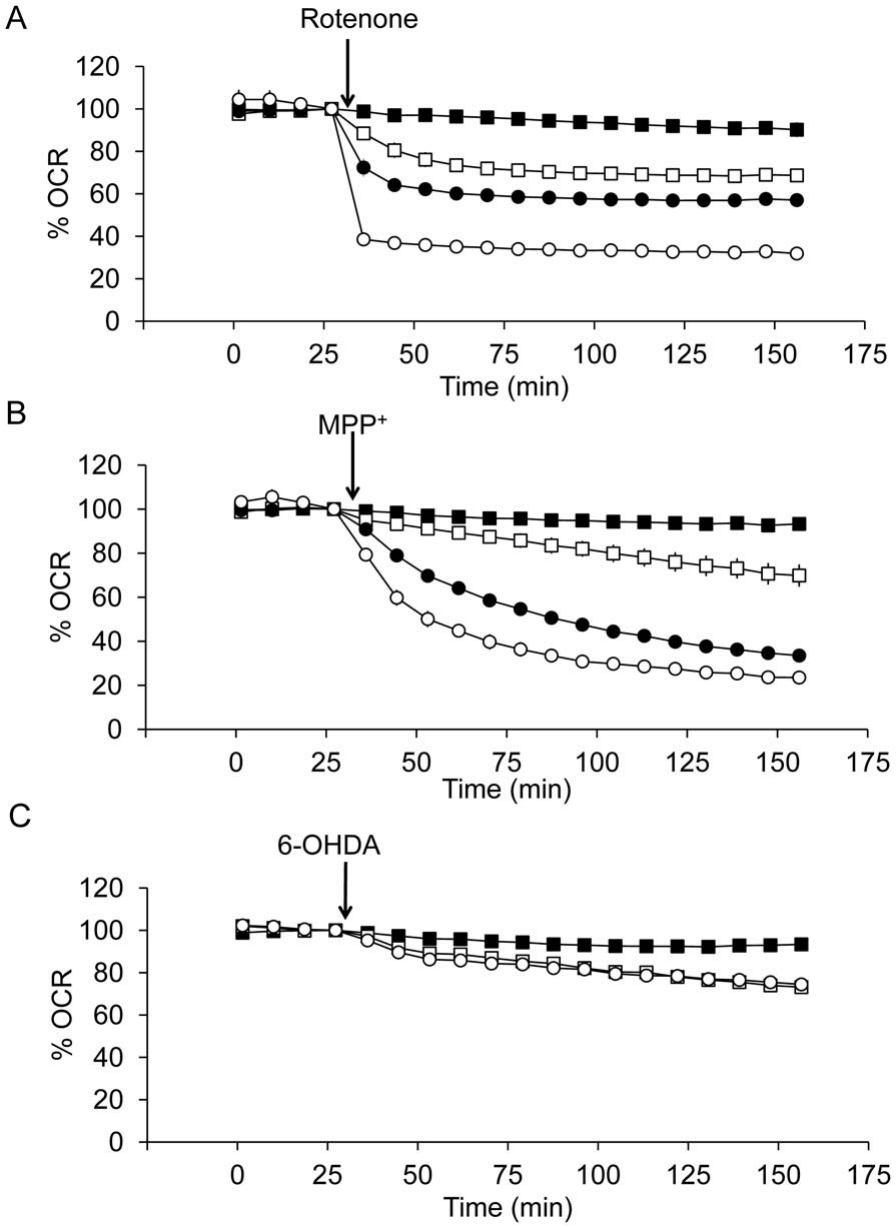
Concentration-dependent effects of rotenone, MPP^+^, and 6-OHDA on basal OCR. Using the XF24 analyzer, the mitochondrial oxygen consumption rate (OCR) was determined for 4 basal readings with 80,000 cells plated per well. OCRs were between 8–12 pmol O_2_/min/µg protein. Then rotenone (A), MPP^+^ (B), and 6-OHDA (C) were injected. (A) ▪ control, □ 1 nM rotenone, • 10 nM rotenone, and ○ 100 nM rotenone. (B) ▪ control, □ 500 nM MPP^+^, •5 µM MPP^+^, and ○ 500 µM MPP^+^. (C) ▪ control, □ 50 µM 6-OHDA, and ○ 200 µM 6-OHDA. Data are expressed as percent of the basal OCR prior to injection of neurotoxins. Data  =  mean ± SEM, n = 3. In some cases, the error bars are smaller than the symbols.

**Figure 3:**
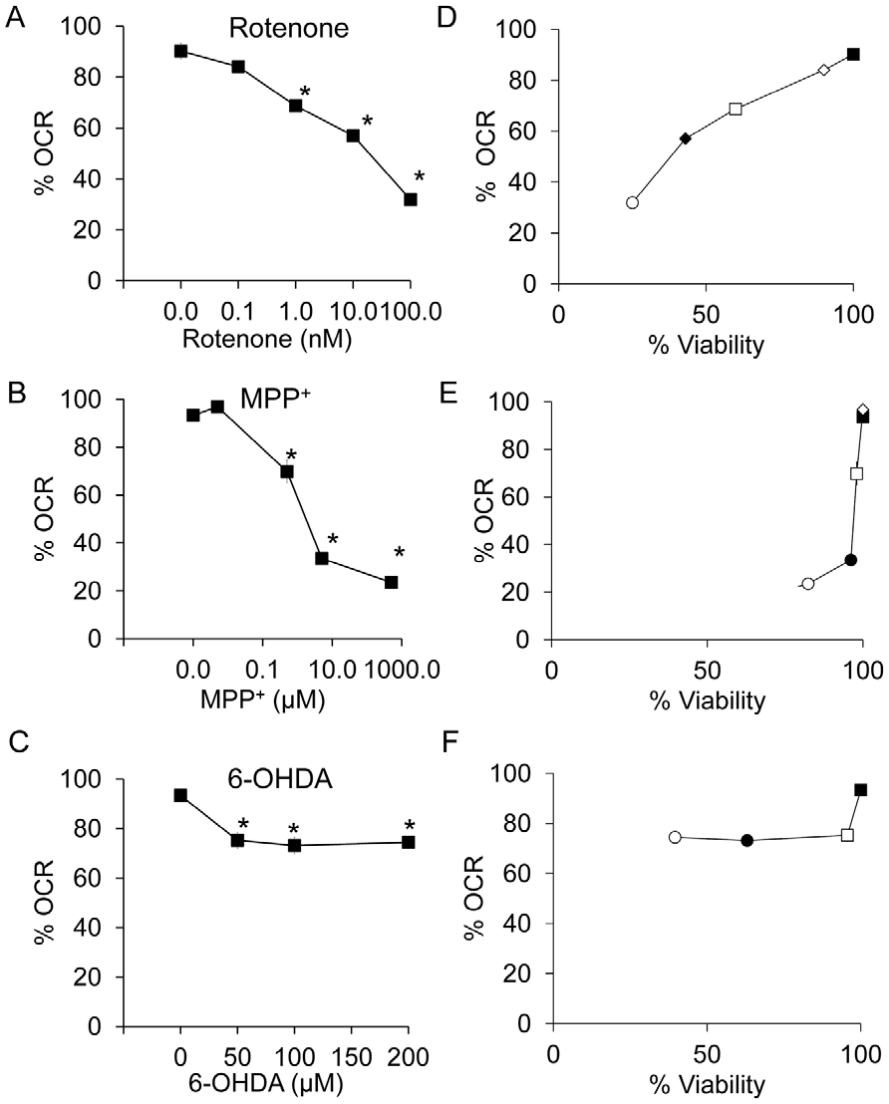
Concentration-dependent effects of rotenone, MPP^+^ and 6-OHDA on basal OCR after 2 hr exposure. Changes in basal OCR after rotenone (A), MPP^+^ (B), and 6-OHDA (C) are shown and are expressed as percent normalized to OCR before injection. Data  =  mean ± SEM, n = 3. *p<0.05, Student *t*-test compared to 0 µM treatment. The relationship between 24 hr % viability and 2 hr % of OCR was plotted for (D) rotenone, (E) MPP^+^ and (F) 6-OHDA. (D) ▪ control, ◊ 0.1 nM rotenone, □ 1 nM rotenone, • 10 nM rotenone, and ○ 100 nM rotenone. (E) ▪ control, ◊ 5 nM MPP^+^, □ 500 nM MPP^+^, • 5 µM MPP^+^, and ○ 500 µM MPP^+^. (F) ▪ control, □ 50 µM 6-OHDA, • 100 µM 6-OHDA, and ○ 200 µM 6-OHDA.

As shown in Figure 2A, 3A, rotenone induced significant effects on basal OCR at concentrations of 1 nM with a rate of onset of inhibition which was dependent on the concentration. MPP^+^ at a concentration of 500 nM or above significantly decreased basal OCR and similar to rotenone the rate of onset of inhibition was dependent on the concentration (Figure 2B and 3B). In contrast, 6-OHDA at concentrations between 50 to 200 µM inhibited OCR to a much lesser extent (<30%), (Figure 2C and 3C). To better understand the relationship between cell viability and mitochondrial function, we have directly plotted the basal OCR versus cell viability by integrating the data from Figure 1B–F and Figure 3A–C. The extent of rotenone induced cell death matches the extent of rotenone induced decrease in OCR. It decreases ∼40% OCR at a dose that decreases ∼50% cell viability. In contrast, MPP+ substantially decreases OCR without substantial decreases in viability. It decreases ∼80% basal OCR at a dose that decreases ∼20% cell viability. 6-OHDA substantially decreases viability without substantial decreases in OCR. It decreases ∼30% basal OCR at a dose that decreases ∼50% cell viability.

**Figure 4:**
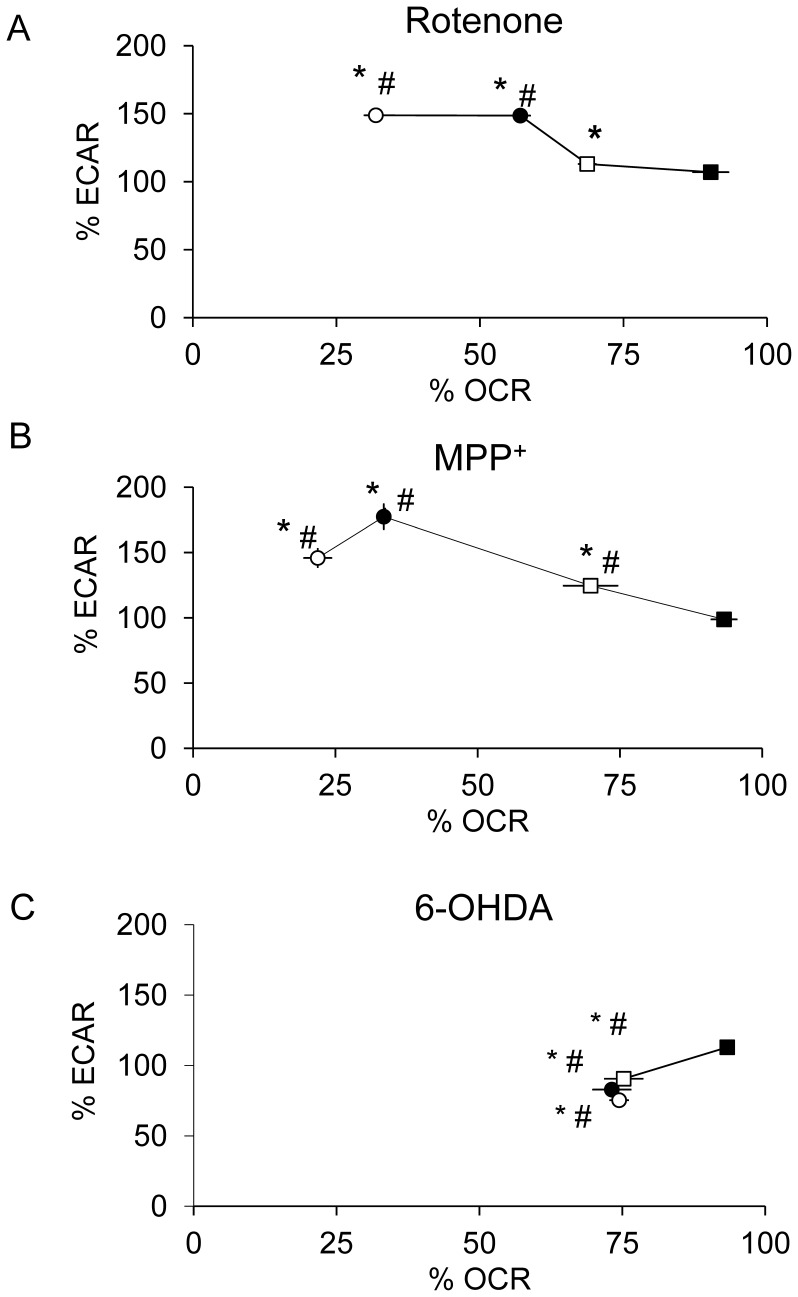
Concentration-dependent effects of rotenone, MPP^+^ and 6-OHDA on OCR and ECAR. The OCR and ECAR data are taken from the 2 hr time point shown in Figure 2 and are expressed as percent normalized to basal OCR or basal ECAR before injection of increasing doses of rotenone (A), MPP^+^ (B), and 6-OHDA (C). ECAR values ranged between 35–110 mpH/min before normalization and 0.5–3 mpH/min/µg protein. (A) ▪ control, □ 1 nM rotenone, • 10 nM rotenone, and ○ 100 nM rotenone. (B) ▪ control, □ 500 nM MPP^+^, • 5 µM MPP^+^, and ○ 500 µM MPP^+^. (C) ▪ control, □ 50 µM 6-OHDA, • 100 µM 6-OHDA, and ○ 200 µM 6-OHDA. Data are expressed as percent normalized to OCR and ECAR before injection. Data  =  mean ± SEM, n = 3. *p<0.05, Student *t*-test compared to 0 µM treatment OCR; #*p*<0.05, Student *t*-test compared to 0 µM treatment ECAR.

Inhibition of mitochondrial respiration stimulates glycolysis and this can be detected by an increase in the rate of extracellular acidification (ECAR). Shown in Figure 4 are the relationships between OCR and ECAR in response to increasing concentrations of the 3 neurotoxins. The data are plotted as % of control for ease of comparison between experiments. Concentrations of rotenone or MPP^+^ which inhibit OCR also increase ECAR to approximately the same extent. There is a biphasic effect of MPP^+^ on ECAR, with maximal stimulation at 5 µM. In contrast, in response to 6-OHDA, both OCR and ECAR are modestly decreased to similar extents.

**Figure 5:**
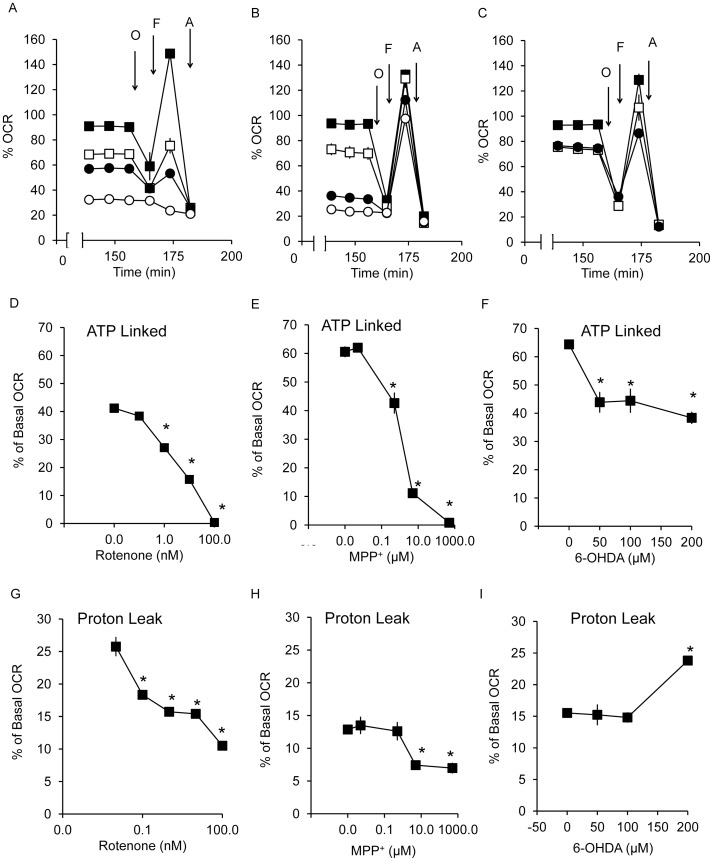
Changes in ATP-linked and proton leak OCR in response to 2 hr exposure to rotenone, MPP^+^ and 6-OHDA. After exposure to rotenone (A), MPP^+^ (B), and 6-OHDA (C) for 2 hr, OCRs were measured after injection of oligomycin (O), FCCP (F) and antimycin A (A). ATP-linked OCR was plotted for rotenone at 0, 0.1, 1, 10 and 100 nM (D), MPP^+^ at 0, 0.005, 0.5, 5 and 500 µM (E), and 6-OHDA at 0, 50, 100 and 200 µM (F); and proton leak OCR for rotenone (G), MPP^+^ (H), and 6-OHDA (I) over the same ranges of increasing doses of neurotoxins as in panels D–F. Data are expressed as percent normalized to OCR before injection of rotenone, MPP^+^, and 6-OHDA. Data  =  mean ± SEM, n = 3. *p<0.05, Student *t*-test compared to 0 µM treatment.

Next we tested the effects of the inhibitors on ATP-linked and maximal respiration using the sequential addition of mitochondrial inhibitors as described previously [69] (Figure 5A–C). The capacity of the respiratory chain to synthesize ATP under basal conditions can be estimated from the extent of decrease in OCR after the addition of oligomycin. The values for ATP linked respiration varied between 40–65% of the Basal OCR depending on the specific cell preparation. We found ATP-linked respiration to be completely inhibited by both rotenone (1–100 nM), and MPP^+^ (0.5–500 µM) in a concentration-dependent manner. In contrast, 6-OHDA (50–200 µM) inhibited ATP-linked respiration to a lesser extent and did not exceed 40% (Figure 5D–F).

The remaining OCR after the addition of oligomycin can be ascribed to proton leak or non-mitochondrial sources of oxygen consumption and varied between 12.5–25% of basal for these preparations. The values for proton leak are shown in Figure 4G–I for the three compounds. Interestingly, rotenone decreased proton leak at 0.1–100 nM, at a concentration lower than that needed to change basal OCR and ECAR. MPP^+^ decreased proton leak at 10–100 µM, whereas 6-OHDA increased proton leak at 200 µM.

**Figure 6:**
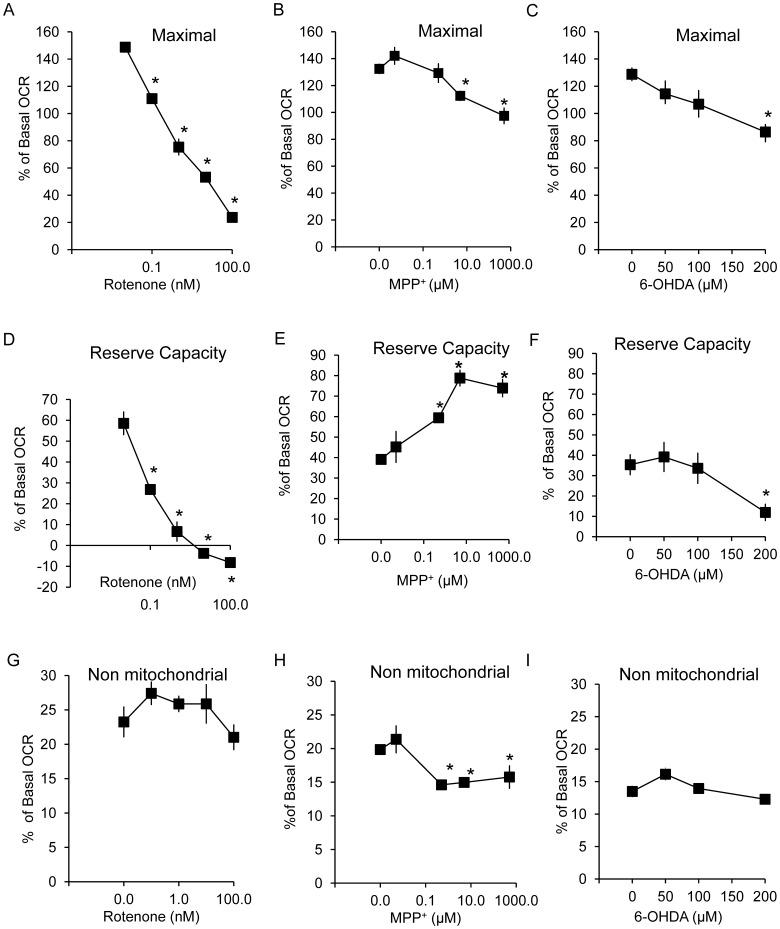
Changes in maximal, reserve capacity and non-mitochondrial OCR in response to 2 hr exposure to rotenone, MPP^+^ and 6-OHDA. Using the OCR traces shown in Figure 5 A–C, maximal OCR, reserve capacity, and non-mitochondrial OCR were determined. Maximal OCR for rotenone (A), MPP^+^ (B), and 6-OHDA (C), reserve capacity for rotenone (D), MPP^+^ (E), and 6-OHDA (F), and non-mitochondrial OCR for rotenone (G), MPP^+^ (H), and 6-OHDA (I) are shown. Data are expressed as percent normalized to OCR before injection of rotenone, MPP^+^, and 6-OHDA. Data  =  mean ± SEM, n = 3. *p<0.05, Student *t*-test compared to 0 µM treatment.

Next FCCP was added to stimulate maximal respiration (Figures 6A–C), and this was also used to calculate the reserve capacity (the difference between basal and maximal OCR) (Figure 6D–F). Interestingly, the behavior of all 3 compounds was markedly different. Rotenone at 0.1–100 nM concentrations inhibited maximal OCR and reserve capacity (Figure 6A,D). MPP^+^ in contrast decreased maximal respiration at 10–100 µM (concentrations that also affect proton leak) with an apparent increase in reserve capacity at 0.5–500 µM (Figure 6B,E). 6-OHDA decreased both maximal OCR and reserve capacity at the highest concentration tested (200 µM) (Figure 6C,F). Non-mitochondrial OCR is unchanged for rotenone and 6-OHDA while is decreased by MPP^+^ at 0.5–500 µM.

## Discussion

Neurotoxin models play an important role in Parkinson’s research and the compounds described here have been used by many researchers with isolated mitochondria, cultured cells and animal models of the disease [15], [16], [45], [70]–[85] . In this study we provide data which bridges the gap between experiments with rotenone, MPP^+^ and 6-OHDA in isolated mitochondria, with cell and animal models by assessment of cellular bioenergetics. To achieve this we have selected a dopaminergic cell line, SH-SY5Y, which has been used extensively as a model to test the effects of neurotoxins on cell function [45], [70]–[85]. We have recently shown that the bioenergetic profile of differentiated SH-SY5Y cells possess many of the hallmarks of neurons including the presence of a bioenergetic reserve capacity [70]. All three neurotoxins have been tested in this cell model in a wide range of studies [45], [72], [75], [76], [80], [81]. The differentiated forms of these cells have an active dopamine transporter although it may have a lower activity than in mesencephalic dopaminergic neurons, it has similar activity to that found in synaptosomes [45], [72], [85]. It is important to recognize that the relative potency may change depending on cell type and the levels of expression of DAT. Primary dopaminergic neurons from rodents may better resemble human dopaminergic neurons in terms of cellular properties used but unfortunately, cellular bioenergetic analysis requires homogeneous cell populations and this is a technical limitation to the use of mesencephalic neurons which are heterogeneous and are not easy to obtain in large numbers [86] .

To gain more insight into the mechanisms of how these neurotoxins affect cellular bioenergetics, we assessed different aspects of the bioenergetic profiles [69]. Oligomycin is an inhibitor of the ATP synthase and when added to the cells will decrease the basal OCR. The extent to which this decreases after a treatment can be ascribed to the inhibition in the cell of an ATP consuming process, inhibition of the ATP synthase or related proteins, or decreased ability of the electron transport chain to provide sufficient proton motive force to drive ATP synthesis. All 3 compounds, rotenone, MPP^+^ and 6-OHDA exhibit a good correspondence between the extents to which they decreased ATP linked respiration and inhibited basal OCR (Figure 5). However, only rotenone and MPP^+^ completely inhibited both basal and ATP linked respiration. The remaining OCR after the addition of oligomycin is ascribed to proton leak. In this parameter the 3 neurotoxins also showed different responses. Rotenone decreased proton leak even at 0.1 nM concentration, whereas MPP^+^ decreased proton leak at 10–100 µM concentrations. In contrast, 6-OHDA increased this parameter at 200 µM. We interpret this result to indicate that rotenone and MPP^+^ may have increased mitochondrial efficiency by decreasing proton leak whereas 6-OHDA has decreased efficiency. We have previously shown that increase ROS can cause an increase in proton leak consistent with the reported pro-oxidant effects of 6-OHDA [87].

Adding the proton ionophore FCCP removes the regulation of the proton motive force on basal respiration, and allows full activity of the respiratory chain to be realized depending on the substrate availability from cellular metabolism. The difference between basal and maximal OCR is termed the reserve or spare bioenergetic capacity and, in the absence of any other bioenergetic defects, can be used to service increased energy demands in the cell including increased oxidative stress [68], [69]. Using this approach, we observed that rotenone exposure decreased maximal respiration and reserve capacity at 0.1 nM, while, in contrast, MPP^+^ increased reserve capacity at 1 µM to 1 mM.

In summary, the response of SH-SY5Y cells to rotenone was as predicted for an authentic complex I inhibitor. Specifically, inhibition of cellular respiration by rotenone results in the compensatory induction of glycolysis, loss of bioenergetic reserve capacity, activation of the apoptotic cascade and a strong correspondence between the doses which cause bioenergetic dysfunction and cell death. In contrast, MPP^+^, while inhibiting basal respiration to a similar extent as rotenone, did not induce the same level of cytotoxicity. This difference could occur because rotenone has an additional effect leading to cytotoxicity independent of inhibition of basal respiration or the mechanism through which MPP^+^ inhibits respiration is different. Our data suggests that MPP^+^ interacts through the respiratory chain in a different manner since basal cellular respiration is inhibited but can still be stimulated by the addition of uncoupler, suggesting an essentially unimpaired electron transport chain and possibly inhibition of ATP synthase or related proteins. The apoptotic pathway is not activated, and the doses which cause inhibition of mitochondrial function do not correspond well to cytotoxic doses. An interesting possibility is that MPP^+^ is acting as a cation, and as such its distribution and interactions will depend on mitochondrial membrane potential [88]. At the present time we cannot exclude the possibility that MPP^+^ levels are decreased by the addition of FCCP but if this were the case inhibition of electron transport which would also decrease membrane potential should result in reversal of its inhibitory effects. Recent gene array studies in SH-SY5Y cells suggest that ATP synthase is down regulated in response to MPP^+^, which would be consistent with a change in both basal and ATP-linked OCR while maintaining reserve capacity [71]. 6-OHDA has none of the characteristics of an inhibitor of mitochondrial oxidative phosphorylation, direct or indirect, and the observed responses to bioenergetics, such as increased proton leak, are consistent with oxidative stress [62], [63], [65]. It is possible 6-OHDA impacts mitochondrial redox signaling without engagement of the major intra-mitochondrial metabolic pathways.

ROS generation by rotenone, MPP+ and 6-OHDA has been proposed to contribute to their toxicity. ROS increase in Parkinson’s disease has been well documented, although clinical trials using antioxidants have not been proven efficacious [89]. Furthermore, a most recent study in the Journal of Neurochemistry [90] has shown that at doses clearly affecting mitochondrial function, there is a lack of correlation with superoxide generation. In this study we further conclude that the widely used experimental neurotoxins, rotenone, MPP^+^ and 6-OHDA, at doses that induce cell death in differentiated dopaminergic SH-SY5Y cells, do not result in similar changes in bioenergetic function.
